# Cellulose Nanofiber-Based Aerogels from Wheat Straw: Influence of Surface Load and Lignin Content on Their Properties and Dye Removal Capacity

**DOI:** 10.3390/biom12020232

**Published:** 2022-01-29

**Authors:** Ramón Morcillo-Martín, Eduardo Espinosa, Laura Rabasco-Vílchez, Laura M. Sanchez, Jorge de Haro, Alejandro Rodríguez

**Affiliations:** 1 Biopren Group (RNM940), Chemical Engineering Department, Faculty of Science, Universidad de Córdoba, 14014 Córdoba, Spain; t62ravil@uco.es (L.R.-V.); laura.sanchez@fulbrightmail.org (L.M.S.); q42hanij@uco.es (J.d.H.); a.rodriguez@uco.es (A.R.); 2 Department of Food Science and Technology, Faculty of Veterinary, Universidad de Córdoba, 14014 Córdoba, Spain; 3 Materiales Compuestos Termoplásticos (CoMP), Instituto de Investigaciones en Ciencia y Tecnología de Materiales (INTEMA), CONICET–Universidad Nacional de Mar de Plata (UNMdP), Mar de Plata 7600, Argentina

**Keywords:** aerogels, dye removal, lignocellulosic biomass, circular economy, biorefinery

## Abstract

Water pollution is one of the most serious problems worldwide. Nanocellulose-based aerogels usually show excellent adsorption capacities due to their high aspect ratio, specific surface area and surface charge, making them ideal for water purification. In this work, (ligno)cellulose nanofibers (LCNFs/CNFs) from wheat straw residues were obtained using two types of pre-treatments: mechanical (Mec) and TEMPO-mediated oxidization (TO), to obtain different consistency (0.2, 0.4, 0.6 and 0.8) bioaerogels, and their adsorption capacities as dye removers were further studied. The materials were characterized in terms of density, porosity and mechanical properties. An inversely proportional relationship was observed between the consistencies of the aerogels and their achieved densities. Despite the increase in density, all samples showed porosities above 99%. In terms of mechanical properties, the best results were obtained for the 0.8% consistency LCNF and CNF-Mec aerogels, reaching 67.87 kPa and 64.6 kPa for tensile strength and Young’s modulus, respectively. In contrast, the adsorption capacity of the aerogels was better for TEMPO-oxidized aerogels, reaching removal rates of almost 100% for the CNF-TO5 samples. Furthermore, the residual lignin content in LCNF-Mec aerogels showed a great improvement in the removal capacity, reaching rates higher than 80%, further improving the cost efficiency of the samples due to the reduction in chemical treatments.

## 1. Introduction

Water pollution is one of the most serious problems worldwide that not only disrupts water supplies but also endangers public health. Among the usual pollutants that can be found in water are nutrients, microbial pollutants, heavy metals and priority pollutants [1]. In recent years, there has been an interest in the study of new methods for the removal of dyes from water due to its large-scale use in different industries (for textile, leather, paper printing, cosmetic, pharmaceutical, food and technological applications) [2]. It is estimated that more than 700 thousand tons of about 10,000 different types of dyes are produced annually, most of which are of synthetic origin and can generate adverse effects (teratogenic, mutagenic and carcinogenic action) on both aquatic organisms and humans [3,4,5]. In general, dyes are mainly applied in the textile industry, and they are usually classified into anionic (acid dyes), cationic (basic dyes) and non-ionic (disperse dyes) dyes. The high solubility of some of these dyes makes their fixation difficult in the clothing dyeing process, considering the rate of dye loss throughout the textile manufacturing processes to be around 10–15%. Thus, this leads to wastewater containing high amounts of this class of organic compound, which contributes to environmental pollution when it is improperly discharged [6]. One of the organic compounds commonly used for dying textiles, wood, paper and plastics is methylene blue [7]. It can cause different diseases in humans and animals, such as permanent damage from eye burns, breathing disorders, heart rate increases, shock, cyanosis, jaundice, quadriplegia, tissue necrosis, nausea, vomiting, mental confusion, painful micturition and methemoglobinemia [8,9].

Over time, different treatments have been used for dye removal, including physical methods, such as filtration, flocculation and irradiation, and other conventional chemical methods, such as advanced oxidation processes, electrochemical degradation or ozonation [1,10,11,12]. The problem with these treatments lies in their affordability and efficiency. Furthermore, chemical treatments produce secondary wastes that need to be treated, and they are more expensive than other techniques because they usually require high amounts of energy and specific equipment to be performed [13]. Therefore, new alternative treatments have been considered for effective dye removal. One of the treatments that has been shown to be promising in water decontamination is adsorption [14]. Specifically, a key parameter for the adsorption of dyes is the choice of the adsorbent [15], which can be diverse, ranging from metal oxides and nitrites to zeolites, clays, polymers and biobased polymers [16,17,18,19,20].

One of the most promising adsorption materials used in water decontamination is aerogels, a class of solid materials featuring a porous structure and an extremely low density. Aerogels are prepared by replacing the liquid phase of a wet gel with a gas, which results in a dry porous solid structure [21]. In general, they contain more than 95% porosity, with an average pore size of 100 nm, a large specific surface area, excellent conductivity, good mechanical properties and high chemical stability [21,22]. In recent years, there has been increasing interest in the use of sustainable resources such as polysaccharides and proteins to produce aerogels (bioaerogels), thus replacing aerogels based on synthetic polymers (such as polyamides, polyimides and polyurethanes) and, as a result, looking forward to the reduction in the environmental impact associated with the polymer industry [23,24,25]. The most abundant renewable resource on Earth, and therefore the largest source of biopolymers, is lignocellulosic biomass. One of the industries that generates waste with a lignocellulosic nature is the agricultural and agri-food industry. The large-scale production mechanization as well as the increase in the world population has led to an increase in the production of this type of waste, and its valorization is considered as essential for the achievement of a correct bioeconomy and sustainable development. One of the most widely grown crops globally is wheat, with an estimated production of more than 776 million tons in 2021 [26]. The abundance and characteristics of wheat straw have allowed its exploitation as a source of cellulose, lignin, bioethanol, bio-insulation, prebiotics, etc. [27,28,29,30,31], highlighting its potential as a source of biopolymers to be valorized in the production of lignocellulosic-based materials through biorefinery processes [32,33,34].

Lignocellulose-based materials, particularly cellulose, are not generally considered for high value-added applications, as these polymers have traditionally been used for the production of low-cost bulk goods such as textiles and paper. Luckily, the perception of cellulose’s potential has changed in recent years due to the development of chemical modifications of cellulose and the use of its derivatives as additives and viscosity modifiers in food, cosmetics, pharmaceuticals and paints, and also as thermoplastic polymers for multiple uses. One of the most interesting derivatives for its application in different sectors is nanocellulose. Nanocellulose can be isolated subjecting cellulosic fibers to different treatments (high-pressure homogenization, grinding, cryocrushing, high-intensity ultrasonic, etc.) for their cell wall delamination to the nanometric scale [35]. Due to the high aspect ratio, specific surface area and surface charge of nanocellulose, it forms hydrogels even at low polymer concentrations (0.2–1%), which, after being dried, will form aerogels. The large surface area and excellent adsorption capacities of this nanometric cellulose, especially those corresponding to cellulose nanofibers (CNFs), make it ideal for water purification.

In this sense, several authors have shown the great performance of CNFs and functionalized CNFs in the formation of pure or hybrid aerogels for chemical contaminant remediation (heavy metal, dye and organic oil removal) [36,37,38]. Specifically, for the removal of methylene blue, being a cationic dye, a high anionic charge on the surface of the CNFs is desired to increase their effectiveness during the removal process. One of the functionalization treatments to increase the anionic charge of CNFs is TEMPO-mediated oxidation. In TEMPO-mediated oxidation treatment, the primary alcohols of cellulose molecules are selectively oxidized into aldehyde and carboxyl groups, reducing the polymerization degree and increasing the surface charge in the fiber [39]. The adsorption of the dye on the surface of CNFs is the result of an ion exchange process between the anionic active sites and the positively charged dye molecules, whereby the structural changes produced during the oxidation of the nanofibers lead to an increase in H bonding, pore filling capacity and electrostatic interaction, enhancing the cationic dye removal process [40].

In this work, cellulose nanofibers were isolated from wheat straw for the development of CNF-based aerogels, and to study their adsorption capacities in the removal of dyes, using methylene blue as a model pollutant.

## 2. Materials and Methods

### 2.1. Materials

The wheat straw residues (WS) used in this work were provided by an independent farmer from Córdoba (Spain). The wheat straw was dried at room temperature to sub-10% humidity and stored in plastics bag after the removal of undesired elements by manual screening. The reagents used in this work were: acetone (Sigma Aldrich, Saint Louis, MO, USA); acetic acid (ACS reagent, ≥99.7%); hydrochloric acid (Sigma Aldrich, 37%); sodium chloride (Sigma Aldrich, >99%); sodium hydroxide (Sigma Aldrich, >99%); poly-DADMAC (BTG, Heidenheim, Germany 0.01 N); Pes-Na (BTG, Heidenheim, Germany 0.01 N); TEMPO, 2,2,6,6-tetramethyl-piperidin-1-oxyle (Sigma Aldrich, 98%); sodium hypochlorite (Panreac, Darmstadt, Germany 10%); sodium bromide (Hoynewell, Muskegon, NC, USA); methylene blue (Panreac, >99%).

### 2.2. Methods

#### 2.2.1. Wheat Straw Cellulose Production

Cellulose fibers were isolated from wheat straw after a soda (NaOH) pulping process. The pulping process parameters were 100 °C, 150 min, 7% sodium hydroxide (over dry matter) and a liquid/solid ratio of 10. Once treated, wheat straw was passed through a Sprout-Bauer refiner and separated by sieving through a netting of 0.14 mm mesh size. The yield of the pulping process was determined by a gravimetric method by comparing the dry weight of cellulose fibers obtained with the dry weight of the raw material used in the process. The elimination of the lignin contained in the fibers was achieved by adding NaClO_2_ in acidified conditions (0.3 g of NaClO_2_ per g of fiber mixed with 2% *v*/*v* of acetic acid) to a 3% (*w*/*v*) fiber suspension for 1 h at 75 °C. This treatment process was repeated during 3 cycles until complete fiber bleaching [27].

The chemical composition of the wheat straw (WS), unbleached pulp (WS-UP) and bleached pulp (WS-BP) was analyzed by the following standards: extractives (Tappi T-204), ashes (Tappi T-211), lignin (Tappi T-203os61), holocellulose (Tappi T-222) and α-cellulose (Tappi T-9m54).

#### 2.2.2. Cellulose Nanofiber Isolation

The different cellulosic fractions were delaminated to produce cellulose nanofibers. Depending on the chemical composition of the initial fibers, a distinction was made between lignocellulose nanofibers (LCNFs), those obtained from WS-UP and cellulose nanofibers (CNFs) obtained from WS-BP. Two different pre-treatments were used to facilitate the delamination of the fibers in the nanofibrillation treatment. For mechanical pre-treatment (Mec), the cellulose fibers were refined for 20,000 revs in a PFI beater according to ISO 5264-2:2002 [41]. For TEMPO-mediated oxidation (TO), cellulose fibers were oxidated with 2,2,6,6-tetramethylpiperidinyl-oxyl at different oxidation degrees (3, 5 and 10 mmol) according to the methodology described by Saito et al. [42]. A 1 wt% suspension of pre-treated fibers was prepared and subjected to a high-pressure homogenization treatment in a PANDA 2000 (Gea Niro Soavi, Düsseldorf, Germany). The nanofibrillation treatment was undertaken in 10 passes through the homogenizer: 4 passes at 300 bars, 3 passes at 600 bars and 3 passes at 900 bars. A 1 wt% cellulose nanofiber suspension in gel form was obtained.

#### 2.2.3. Cellulose Nanofiber Characterization

The nanofibrillation yield (ɳ) of the cellulose nanofiber was determined by centrifuging a 0.1 wt% suspension at 12,000 rpm for 10 min to separate the non-nanofibrillated fraction from the nanofibrillated material. The non-nanofibrillated fraction was collected and dried at 100 °C for 24 h to determine the nanofibrillation yield following the methodology described by Besbes et al. [43]. The cationic demand (CD) of the cellulose nanofibers was determined using a particle charge detector, Mütek PCD 05, following the methodology proposed by Carrasco et al. [44] but adapted for cellulose nanofibers [41]. The carboxyl content (CC) of the cellulose nanofibers was analyzed by conductometric titration following the protocol described by Saito et al. [45]. The specific surface area and diameter of cellulose nanofibers were theoretically determined from the net cationic demand (CD–CC) following the premises and methodology proposed by Carrasco et al. [44]. The intrinsic viscosity of the cellulose nanofibers was determined according to UNE 57-039-95 and used for the estimation of the degree of polymerization (DP), as described by Marx-Figini [46]. The DP indicates the number of glucose monomers forming the cellulose chain; therefore, this parameter is related to the length of the cellulose chain, as proposed by Shinoda et al. [46].

The main functional groups of the different samples were identified by Fourier transform infrared spectroscopy (FTIR) using a spectrometer, FTIR-ATR Perkin-Elmer Spectrum Two. The samples were analyzed in the range of 450–4000 cm^−1^, at a 4 cm^−1^ spectral resolution, with 40 scans.

X-ray diffraction (XRD) data for the different samples were collected using a Bruker D8 Discover with a monochromatic CuKα1 source over an angular range of 5–50° at a scan speed of 0.026°/s. The samples were tested as freeze-dried CNF powder using an adequate sample holder. In order to study the changes in cellulose structure by both TEMPO and mechanical pre-treatments, as well as fiber bleaching, the crystallinity index (*CI*) was estimated according to the Segal method [46] using the following equation:

$$CI\left(\%\right)=\frac{I_{002}-I_{am}}{I_{002}}\cdot 100$$

where *I*_002_ refers to the maximum intensity of the (002) crystallographic plane at 2θ = 22.2°, and *I_am_* is the diffraction intensity for the amorphous cellulose at 2θ = 18.5°.

#### 2.2.4. Cellulose Nanofiber-Based Aerogel Production and Characterization

Different consistencies (0.2, 0.4, 0.6 and 0.8 wt%) of CNF suspensions were prepared after diluting the original suspension with defined amounts of distilled water and subsequently homogenized at 10,000 rpm for 8 min with an Ultraturrax IKA T-18 system. Each suspension was freeze dried at −85 °C under 0.5 mBar for 72 h in a Lyoquest −85 device. In order to ensure that only sublimation occurred, suspensions were frozen for 24 h prior to freeze drying.

The aerogels’ apparent density (*ρ* = mass/volume, in kg/m^3^) was obtained by the cylinder volume method using a digital caliper (0.01 mm accuracy) and digital balance (0.0001 g accuracy). Since porosity is a key property in the formation of aerogels due to its relationship with adsorption capacity [47], aerogels’ porosity was calculated using the following equation:

$$Porosity\;\left(\%\right)=\left(1-\frac{\rho_{sample}}{\rho_{cellulose}}\right)\cdot 100$$

where ρ_sample_ is the aerogel density expressed in kg/m^3^, and ρ_cellulose_ is the bulk density of cellulose, taken as 1500 kg/m^3^.

The mechanical properties of the aerogels were tested using a universal testing machine (Model LF Plus Lloyd Instrument9) equipped with a load cell of 1 kN. The aerogel samples were cut into cylinders approximately 12 mm in height and 25 mm in diameter, and they were placed in a pair of fixed plates 110 mm in diameter. The mechanical properties of the aerogels were evaluated by compression tests with a strain limit of 80%, based on the initial aerogels’ height, at a speed of 2 mm/min.

#### 2.2.5. Adsorption Study of Methylene Blue with Cellulose Nanofiber-Based Aerogel

Batch adsorption experiments were carried out by immersing 100 mg of aerogel adsorbent in 50 mL of methylene blue (*MB*) solution at *MB* concentrations of 5 and 20 mg/L under constant magnetic stirring at ambient temperature for 24 h. At fixed time intervals, the concentration of *MB* solution was measured at 665 nm using a UV–Vis spectrophotometer, Shimadzu UV-160A. The amount of methylene blue adsorbed at each time interval on the cellulose nanofiber-based aerogels was calculated as

$$MB\;removal\;\left(\%\right)=\frac{C_{0}-C_{t}}{C_{0}}\cdot 100$$

where *C*_0_ is the initial methylene blue concentration, and *C_t_* is the methylene blue concentration at time *t*.

## 3. Results

### 3.1. Cellulosic Fiber Chemical Characterization

Table 1 shows the results of the fiber chemical characterization for WS, WS-UP and WS-BP. In general, the typical composition of a lignocellulosic material is observed to have cellulose, hemicelluloses and lignin as the main components. The cellulose content of WS was similar to that found in other non-woody feedstocks, such as barley, rapeseed and oats, with 34%, 37% and 38%, respectively [48]. This justifies the potential of this raw material to produce cellulose pulp and cellulose nanofibers.

After the pulping process, the non-structural components of the raw material such as extractables and ashes were reduced. For WS-UP, the cellulosic fraction was increased from 33% to 51%, while the hemicellulose content was maintained in the fiber with values close to 25%. Retaining a high content of hemicelluloses in the fibers is of special interest in cellulose nanofiber production because they act as a barrier to microfiber aggregation during the homogenization process [49]. The bleaching process oxidizes lignin from the fibers, while the cellulose and hemicellulose contents remain intact. As shown in Table 1, the bleaching process led to a decrease in the lignin content from 9.9% to 2.5%, while maintaining and increasing the hemicellulose and cellulose contents, respectively. This step facilitates the accessibility of functional groups, making the fibers more amenable to functionalization by pre-treatments, thus favoring their subsequent nanofibrillation [27].

### 3.2. Cellulose Nanofiber Characterization

Table 2 shows the results of the nanofibrillation yield (ɳ), cationic demand (CD), carboxyl content (CC), specific surface area (σ_spec_), length, diameter and aspect ratio determinations for the different LCNFs and CNFs obtained from the two types of pre-treatments, mechanical (Mec) and TEMPO-mediated oxidation (TO). In the case of TO, three oxidation degrees, 3, 5 and 10 mmoles of NaClO per gram of fiber (TO3, TO5, TO10), were used.

Overall, the characterization results were promising for all types of nanofibers, showing similar or even better values than other raw materials [51,52]. However, we can observe that the results for the mechanically obtained nanofibers were not as good as those obtained with the TEMPO pre-treatment. A possible explanation for this fact lies in the high stability of the cellulose microfibrils. They possess numerous inter-fibrillar hydrogen bonds that confer high cohesion, making it difficult to completely individualize the microfibrils by mechanical processes, resulting in a less efficient nanofibrillation process [53]. Moreover, this type of pre-treatment does not depolymerize the cellulose fibers as much as TEMPO, resulting in longer fiber lengths, reaching values of 4121 and 4224 nm for CNF-Mec and LCNF-Mec, respectively. The presence of residual lignin in the fibers leads to lower diameters in the production of cellulose nanofibers by mechanical pre-treatment. The same behavior was previously observed by Ferrer et al. [54]: the presence of residual lignin and other cell wall components in birch fibers induced the formation of thinner fibrils after mechanical shearing [55]. Then, the antioxidant effect from this residual lignin could prevent the re-bonding of the previously broken covalent bonds.

Regarding the cationic demand and carboxyl content, they were lower in mechanically treated samples compared to nanofibers obtained by chemical pre-treatment. The increase in the carboxyl content from nanofibers obtained by TEMPO-mediated oxidation is due to the oxidative process itself, in which C6 primary alcohols of cellulose are converted into carboxyl groups. On the other hand, the higher results for the cationic demand are explained by the synergistic effect between TEMPO pre-treatment and the better efficiency in the nanofibrillation process, resulting in a larger exposed surface area and therefore higher surface charge, facilitating the homogenization step.

Focusing on the different results obtained for CNF-TO and LCNF-TO, it is noted that the lignin content negatively affects the TEMPO pre-treatment. The presence of lignin interferes with the reaction activator (NaClO), which is also consumed as a bleaching agent, producing the oxidation and dissolution of lignin. A direct relationship between the oxidative power of the TEMPO pre-treatment and the results for the cationic demand, carboxyl content and specific surface area of the fibers can be observed. The best results were obtained for CNF-TO10 nanofibers, reaching values of 96.4%, 1440 μeq/g, 369.5 μeq/g and 5 nm for ɳ, CD, CC and diameter, respectively. However, these values are not as good when considering LCNF-TO10, showing a decrease in the efficiency of TEMPO oxidation in fibers with residual lignin. The intensity of the TEMPO pre-treatment had a great influence on these parameters. It can be concluded that applying a 5 mmol TEMPO pre-treatment is sufficient to achieve the highest values of nanofibrillation yields, carboxyl contents and diameters. Further oxidation in the pre-treatment may not be worthwhile, due to the good nanofibrillation yields and nanometric diameters obtained for milder pre-treatments. Additionally, a further increase in the oxidation intensity produces a significant degradation in the fiber, as seen in the shortening of the fiber, without sufficiently improving the rest of the properties. A higher efficiency of the TEMPO-mediated oxidation pre-treatment on lignin-free fibers can be observed, allowing greater accessibility to primary alcohols for conversion into carboxyl groups when lignin is not contained.

Although not of relevance for methylene blue removal by adsorption, the thermal stability of cellulose nanofibers may also be of interest if the aerogels produced are used in applications that require subjecting such materials to high temperatures. Previous studies showed that these materials exhibit the typical thermal degradation stages of lignocellulosic materials (i. moisture loss (50–120 °C); ii. thermal degradation of hemicelluloses (250–400 °C); and iii. pyrolysis of certain cellulose and lignin compounds (400–800 °C)), showing T_max_ (maximum degradation temperature) values in the 300–340 °C range. No significant differences were observed in thermal stability relative to the presence of lignin, but a higher carbonaceous residue was observed after pyrolysis of the lignocellulosic components due to the aromatic structure of lignin [50,51].

#### 3.2.1. FTIR Analysis

**Figure 1:**
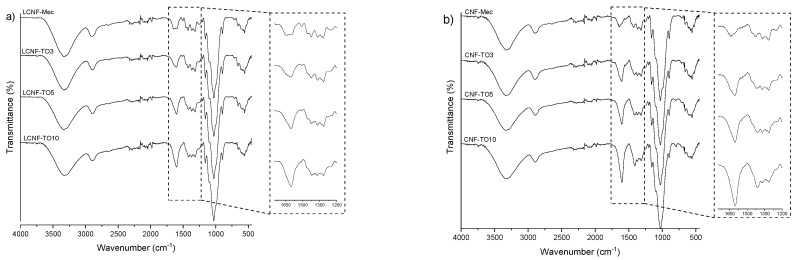
ATR-FTIR spectra of lignocellulosic nanofibers (LCNFs) (**a**), and cellulose nanofibers (CNFs) (**b**).

Figure 1a,b show the spectra obtained from the FTIR analysis of the different LCNFs and CNFs obtained in this work.

All samples showed the typical spectrum of a lignocellulosic material. The stretching vibrations of the OH groups can be observed around 3330 cm^−1^, while the C=O stretching of the carboxyl groups occurs around 1600 cm^−1^. The next peak detected in the spectra is around 1502 cm^−1^, corresponding to the aromatic ring of lignin. Peaks around 1420 cm^−1^ correspond to CH_2_ groups. Finally, around 1030 cm^−1^, peaks related to the carbonyl bonds present in the cellulose skeleton can be observed.

If we focus on the LCNF spectra (Figure 1a), specifically on the 1600 cm^−1^ peak corresponding to the carboxyl groups, we can see that in the TO10 pre-treatment, this peak is notoriously higher compared to the TO3, TO5 and Mec pre-treatments. This is due to the higher oxidation intensity resulting in the generation of a higher number of carboxyl groups (366.6 µeq/g), as shown in Table 2. However, if we compare this same peak with that corresponding to the CNF spectra in Figure 1b, we can see that the peaks corresponding to CNFs are slightly higher. This increase in the peaks confirms the greater effectiveness of the TEMPO pre-treatment on lignin-free fibers, avoiding the undesired oxidation of lignin during the treatment, using the full oxidative capacity of the selective oxidation of cellulose. Focusing on the two types of pre-treatments, we can see that the LCNF-Mec spectrum shows a higher intensity of the peak around 1502 cm^−1^ (corresponding to the aromatic ring of lignin) compared to the chemically pre-treated samples. This shows that although the TEMPO reaction performs a selective oxidation of cellulose, partial oxidation of lignin also takes place. The lignin peak is absent in the CNF spectra (Figure 1b), confirming the residual value of lignin after the bleaching process. Furthermore, a higher efficiency of TEMPO-mediated oxidation can be observed in the lignin-free fibers, where the TO3 pre-treatment is enough to return a carboxyl peak intensity similar to that in the LCNFs with a higher-oxidation degree treatment.

#### 3.2.2. XRD Analysis

**Figure 2:**
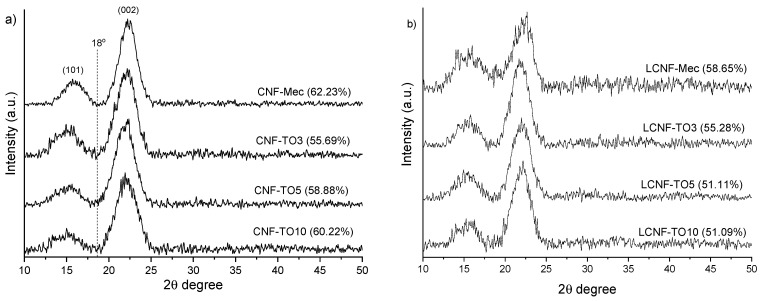
XRD patterns and crystallinity index for lignocellulosic nanofibers (LCNF) (**a**), and for cellulose nanofibers (CNF) (**b**).

The crystalline structure of the different CNFs and LCNFs was analyzed by XRD. As shown in Figure 2, two crystallinity peaks were obtained at 2θ = 16° and 22.5°, which were typical cellulose I peaks [56,57]. It can be observed that the crystallinity index (CI) of cellulose nanofibers from bleached fibers was higher than that obtained from unbleached fibers, due to the reduction in or elimination of the amorphous non-cellulosic compounds (hemicellulose and lignin) present in the fibers as a result of the bleaching treatments, as confirmed by both the chemical characterization and the FTIR analysis. The crystalline domains of cellulose were affected by each pre-treatment. The decrease in the CI in TEMPO-oxidized cellulose could be explained by the fact that almost all carboxyl groups formed during oxidation are present on the surfaces of the crystalline cellulose microfibrils [58] and by the sodium glucuronosyl units, leading to the conversion of some crystalline regions of cellulose into disordered structures during the oxidation reaction [59]. However, when overoxidation occurred in the high-oxidation TEMPO pre-treatments, some non-crystalline parts were degraded, thus shortening the fibers and causing an increase in the crystallinity of the fibers, as occurred in the CNFs. For the LCNFs, this was not observed. This may be because the overoxidation conditions in LCNFs produce a non-selective oxidation of the lignin (dissolving it and bleaching the fiber), and not a degradation of the cellulose structure.

### 3.3. Cellulose-Based Aerogel Characterization

**Figure 3:**
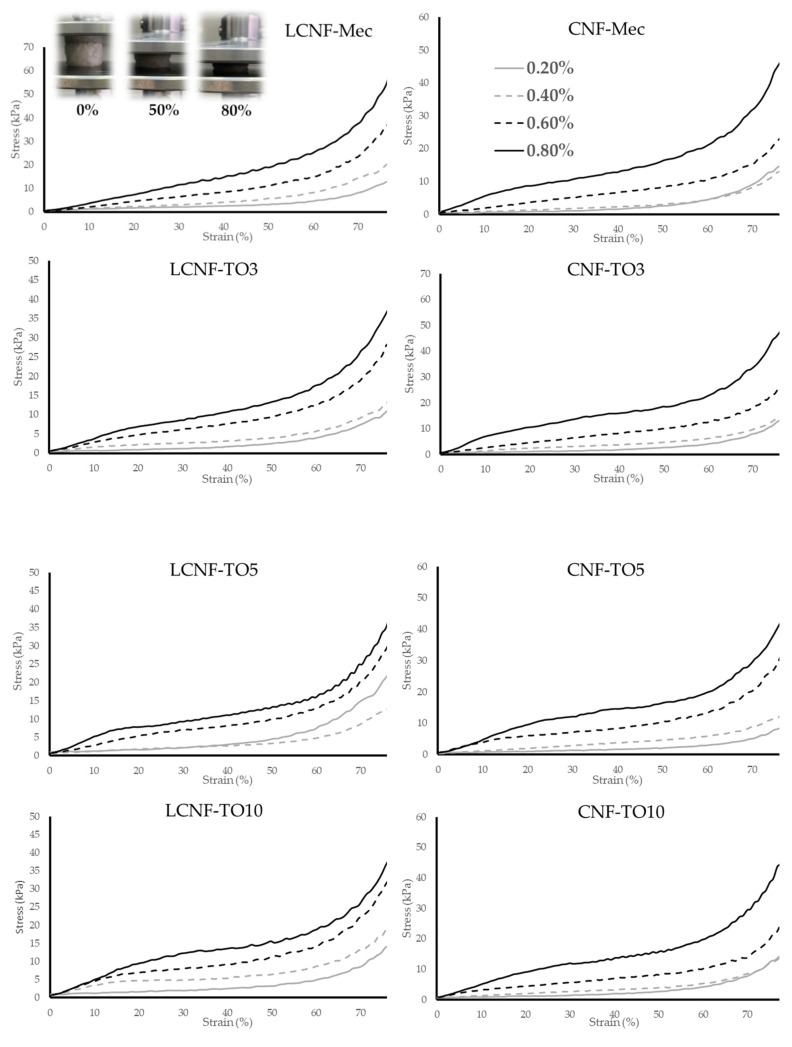
Stress–strain curves from the compression analysis of the different aerogels.

Good mechanical properties are crucial for the handling, performance, regeneration and recyclability of adsorbent aerogels. For this purpose, the stress–strain curves of different aerogels prepared from different CNF/LCNF initial dry solid content suspensions (0.2%, 0.4%, 0.6% and 0.8%) were investigated by means of compression tests (Figure 3). The density of CNF-based aerogels has a direct relationship with the strength and compressive modulus [60]. Sehaqui et al. observed that CNF-based aerogels from 15 to 105 kg/m^3^ resulted in an increase in Young’s modulus from 34.9 to 2800 kPa, and in tensile strength from 3.20 to 238 kPa. As expected, it was observed that when the initial solid concentration was higher, the density of CNF-based aerogels increased, rising from 4 to 16 g/cm^3^ when the consistency was incremented from 0.2 to 0.8% (Table S1). Despite the increase in density, all aerogels showed porosities above 99%, showing a slight reduction with increasing density (Table S1). These results were similar to those obtained by Zhang et al. [61], where they reported cellulose nanofiber aerogels with an average porosity of 98%, showing removals above 90%. However, regardless of the density, all aerogels showed similar stress–strain curves where three clearly differentiated stages could be observed. The first stage (up to 10–15% strain) is the linear elastic deformation of the cellulose structure when subjected to low stresses (<10 KPa). This behavior might be attributed to the elastic deformation of the cell walls and macropore collapse [62]. Then, the curve undergoes a flattening between 15 and 60% with slowly increasing stress due to plastic yielding of the cell walls. In the third region (between 60% and 80% strain), a dramatic increase in the stress can be observed due to the densification caused by the bending or damage of the mesopores as well as the compression and breakage of the covalent bonds between the fibers [63,64,65]. This type of behavior in compression analysis has been reported by other authors [56,57,66]. The stress–strain curves show that the strength values increased considerably when consistencies were higher than 0.4%, obtaining the best results at the highest consistency. For LCNF-Mec and CNF-Mec, values of 67.87 KPa and 61.50 KPa were observed for tensile strength (TS), respectively (Table S1). These results are substantially higher than those obtained by Do et al. [67], in which aerogels made from TEMPO-oxidized nanocellulose reinforced with chitosan showed a maximum YM of 27.25 KPa. Furthermore, the compressive modulus of Mec aerogels was higher than that resulting from a mixture of PVA/carboxymethyl cellulose (0.85–15.42 kPa) [68] and similar to that for CNF aerogels obtained by Huang et al. and Luo et al. [69,70]. Focusing on aerogels prepared from TEMPO-oxidized nanocellulose, better mechanical properties were observed for the CNFs. For both pre-treatments, it could be observed that the higher efficiency in the delamination process of the cellulose nanofibers, and therefore the higher nanofibrillation yields and smaller diameters of the obtained nanofibers, had a large influence on the mechanical properties of the derived aerogels. LCNF-Mec showed better properties than CNF-Mec due to the antibonding effect of lignin during the nanofibrillation process, thus resulting in aerogels with higher mechanical properties. However, LCNF-TO showed worse results due to the interference of lignin with the primary activator (NaClO) of the oxidation reaction, resulting in aerogels with worse mechanical properties than those obtained with CNF-TO. For both cellulose nanofibers, CNFs and LCNFs, it was shown that overoxidation conditions (TO10) resulted in aerogels with worse mechanical properties than those obtained with milder oxidations. This may be due to excessive fiber degradation resulting in shorter fibers with a lower aspect ratio, which directly affects the ability of the fibers to interact with each other, resulting in three-dimensional networks with fewer bonds. As demonstrated, the density has a direct relationship with the mechanical properties of the cellulose nanofiber-based aerogels; therefore, to eliminate this variable in the mechanical property analysis, the specific mechanical properties of the aerogels were calculated (Table S2). In general, it is observed that the specific properties of aerogels do not show a direct relationship with the initial concentration of solids, and thus it is concluded that this parameter does not improve the mechanical properties of the aerogels, beyond the absolute effect that the density has on them.

### 3.4. Cellulose-Based Aerogel Adsorption Capacity

**Figure 4:**
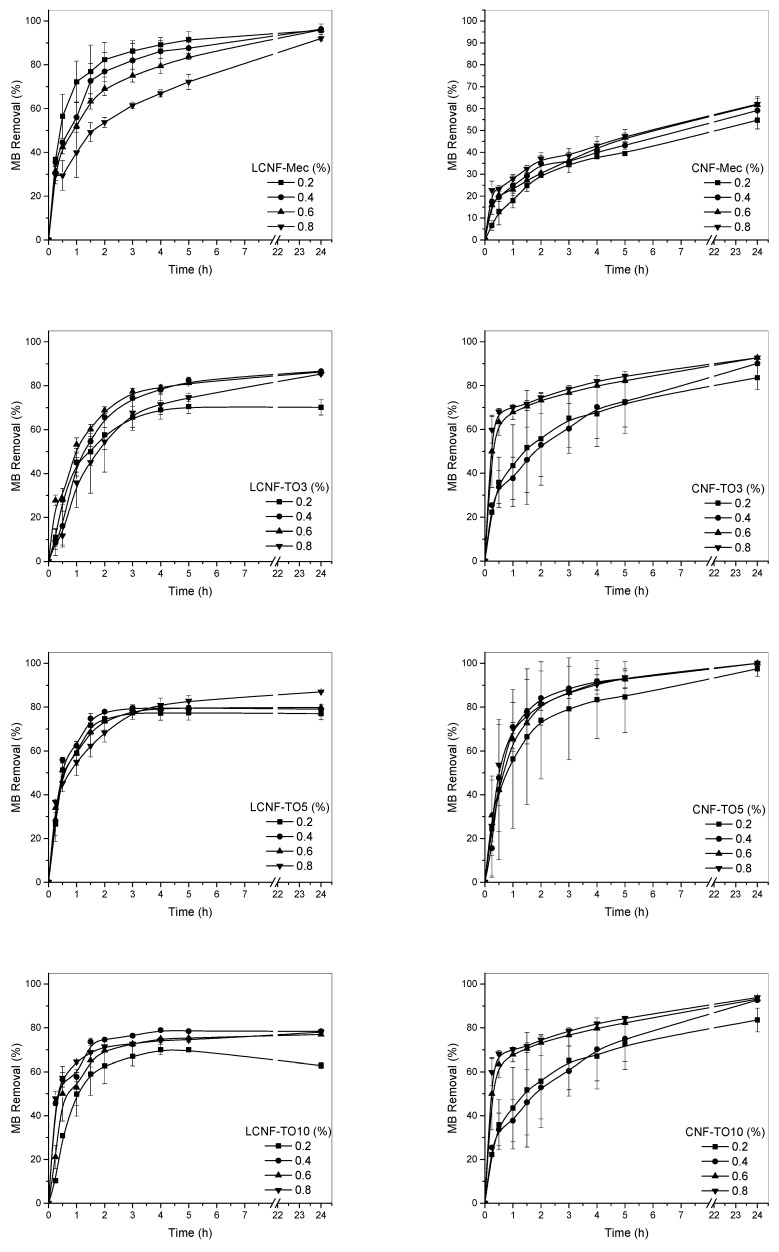
Methylene blue (5 mg/L) adsorption kinetics for the different aerogels.

**Figure 5:**
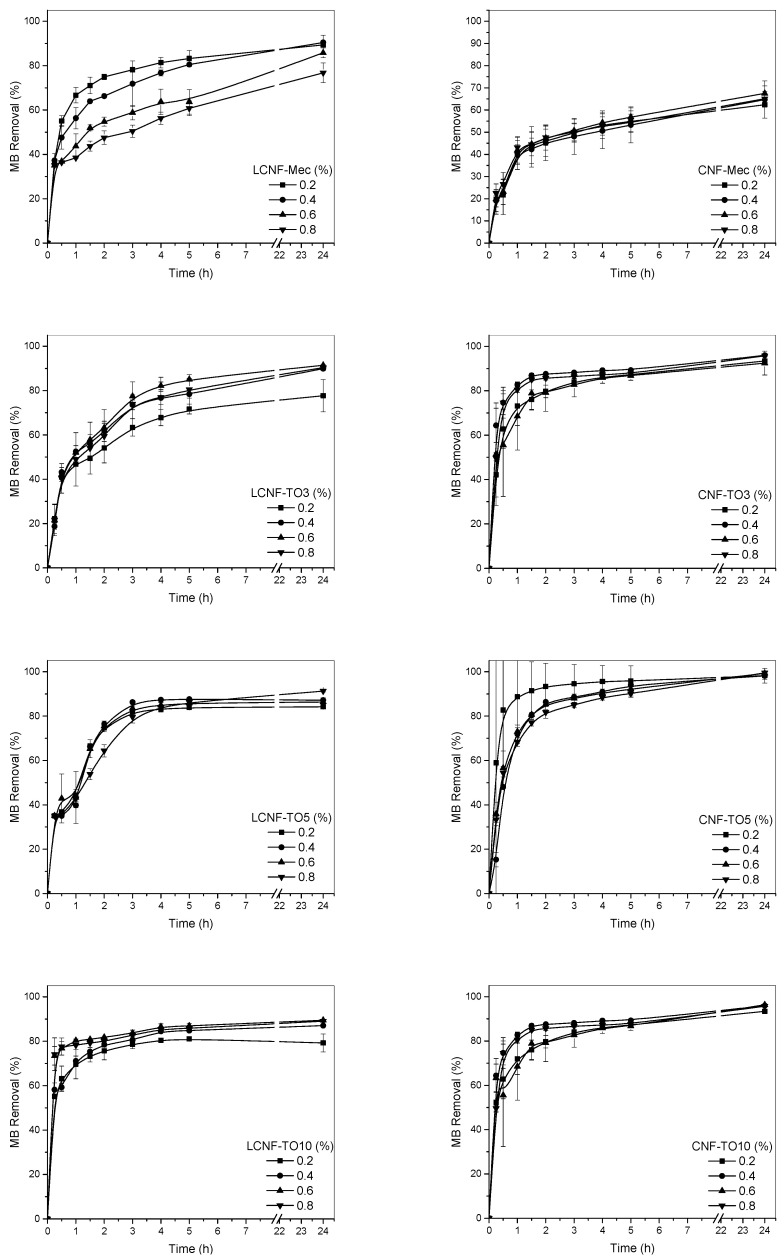
Methylene blue (20 mg/L) adsorption kinetics for the different aerogels.

The adsorption capacity results of the aerogels for the different contaminant concentrations studied are shown in Figure 4 and Figure 5.

In general, two phases are observed in the adsorption curves. The first phase describes an exponential dye removal due to the high availability of hydroxyl and carboxyl groups present in the aerogel (negative charge) to interact with methylene blue (positive charge). At around 2 h, this withdrawal becomes constant due to the lower availability of groups, resulting in a flattening of the curve. The effect of the initial concentration of solids in the production of aerogels on the adsorption capacity of MB was studied for the different samples; however, no significant differences were observed, except for the cases of CNF-Mec and LCNF-Mec. This may be due to the fact that the porosity and internal structure of these aerogels are compromised as the initial solid content, and therefore their density, increases, which does not occur in the aerogels obtained from CNF-TO and LCNF-TO due to the higher surface charge and electrostatic repulsion capacity that give them greater stability in aqueous media, maintaining the porous and three-dimensional structure of the aerogels. The dye removal was slower for aerogels prepared from nanofibers obtained by mechanical treatment compared to TEMPO-oxidized cellulose nanofiber aerogels. The higher surface charge of these aerogels, mainly due to the increase in carboxyl groups and the higher specific surface area found for these cellulose nanofibers, leads to a higher affinity and interaction with methylene blue, obtaining a faster adsorption than that shown by nanofibers obtained by mechanical pre-treatment. Regarding the pollutant concentration, an increase in the initial concentration of methylene blue resulted in a faster adsorption of MB, needing 3 h for 80% dye removal and 2 h for the same dye removal performance for concentrations of 5 mg/L and 20 mg/L. This phenomenon can be explained due to the increase in the driving force of the concentration gradient [71]. The lignin content had a significant effect on the adsorption properties of aerogels derived from nanofibers obtained by mechanical pre-treatment, reaching removal values of 80–90% for LCNF-Mec, similar to the values obtained by TEMPO-oxidized cellulose nanofiber aerogels, and decreasing to 50–60% for CNF-Mec. This is because lignin contains a high concentration of functional groups that can form electrostatic interactions with a wide variety of compounds present in wastewater, including MB [72]. Thus, the obtained results demonstrate the existence of a synergistic effect of nanocellulose and lignin in their use as materials with a high adsorption capacity. This effect was not observed in cellulose nanofiber-based aerogels obtained by TEMPO-mediated oxidation, where the removal of LCNF aerogels was very similar to that of CNF aerogels. However, the high efficiency of both types was evidenced. Similar adsorption curves were reported by Luo et al. [70], where a higher initial concentration of MB resulted in better performances. Comparable results were also observed by Hosseini et al. [73], reaching almost 100% removal after 100 min. Among TEMPO-oxidized nanofibers, TO5 showed the best results, reaching removal rates of almost 100% for CNF-TO5. On the other hand, a decrease in the adsorption capacity was found when increasing the oxidative power to 10 mmol, since no differences were observed in the surface charge of the fibers due to the lack of hydroxyl groups susceptible to be oxidized, but this power does produce degradation in the fibers that can affect their performance as an adsorbent.

## 4. Conclusions

In general, nanocellulose-based aerogels show an excellent adsorption capacity due to their high aspect ratio, specific surface area and surface charge, which makes them ideal for water purification. In this work, cellulose nanofibers (CNFs) and lignocellulose nanofibers (LCNFs) were isolated from wheat straw for aerogel production, and to further study their adsorption capacities in dye removal from model aqueous pollutant solutions. The effect of the applied pre-treatment (mechanical and TEMPO-mediated oxidation, the latter under different operational parameters) on the final properties of the nanofibers was analyzed, and it was found that the efficiency of the different pre-treatments was largely influenced by the initial fiber composition. The physical and mechanical properties of the produced aerogels showed a direct relationship with the initial solid concentration employed: the density and mechanical strength values increased from 4 to 16 g/cm^3^ and from 16 to 67 kPa, when the consistency was increased from 0.2 to 0.8%, respectively. Regarding the contaminant adsorption capacity, the removal of methylene blue was slower in aerogels prepared from nanofibers obtained by mechanical pre-treatment compared to TEMPO-oxidized nanofibers. This can be explained in terms of the high surface charge observed in the aerogels prepared from TEMPO-oxidized nanofibers. On the other hand, although the residual lignin content in LCNF-based aerogels resulted a significant increase in the adsorption capacity of LCNF-Mec aerogels compared to CNF-Mec, similar values were reached by using both types of TEMPO-oxidized nanocellulose nanofibers. The high concentration of lignin functional groups that can interact with contaminants, together with the high adsorption capacity of cellulose nanofibers, demonstrated a synergistic effect in their use as materials with a high adsorption capacity. This article provides information that could be used to create useful, environmentally friendly, cost-effective and efficient adsorbents for the removal of cationic dyes in different wastewater industries. However, further research needs to be performed in order to establish and implement processes for the reuse of these materials, which is relevant to ensure a fully sustainable use of these materials.

## Figures and Tables

**Table 1:** Cellulose fiber characterization for wheat straw (WS), wheat straw unbleached pulp (WS-UP) and wheat straw bleached pulp (WS-BP).

Sample	Extractables (%)	Lignin (%)	Hemicelluloses (%)	α-Cellulose (%)	Ashes (%)
WS	19.8 ± 0.6	15.7 ± 1.7	29.8 ± 0.5	33.4 ± 0.4	7.2 ± 0.1
WS-UP	12.0 ± 0.7	9.9 ± 0.8	24.9 ± 0.2	50.9 ± 0.3	3.7 ± 0.0
WS-BP	7.1 ± 0.8	2.5 ± 0.2	23.5 ± 0.3	62.7 ± 3.9	2.86 ± 0.0

**Table 2:** Characterization of cellulose nanofibers (CNFs) and lignocellulose nanofibers (LCNFs) obtained from mechanical (Mec) and TEMPO-mediated oxidation (TO) pre-treatments.

Pre-Treatment	Sample	ɳ ^1^ (%)	CD ^2^ (μeq/g)	CC ^3^ (μeq/g)	σ_spec_ (m^2^/g)	Length ^4^ (nm)	Diameter (nm)	Aspect Ratio
Mec	CNF	27.1 ± 5.3	328.7 ± 37.2	<74.4	124	4121	20	206
	LCNF	55.6 ± 4.1	441.1 ± 7.4	<74.4	179	4224	14	301
TO3	CNF	85.6 ± 0.0	1160.8 ± 20.0	330.9 ± 1.3	404	1907	6	318
	LCNF	68.8 ± 0.8	728.6 ± 70.9	359.3 ± 1.0	180	1962	14	140
TO5	CNF	89.1 ± 1.5	1210.0 ± 15.5	359.9 ± 0.1	414	1563	6	260
	LCNF	87.6 ± 0.1	925.7 ± 19.8	360.4 ± 0.3	275	1238	9	138
TO10	CNF	96.4 ± 0.4	1440.1 ± 20.1	369.5 ± 2.9	521	1033	5	207
	LCNF	88.4 ± 1.1	1136.3 ± 59.9	366.6 ± 0.7	375	905	7	129

^1^ ɳ refers to the nanofibrillation yield. ^2^ CD stands for the cationic demand of the nanofiber. ^3^ CC refers to the carboxyl content of the sample. ^4^ Calculated from intrinsic viscosity and degree of polymerization [50].

## Data Availability

The data presented in this study are available on request from the first author.

## Supplementary Materials

The following supporting information can be downloaded at: https://www.mdpi.com/article/10.3390/biom12020232/s1, Table S1. Results in Density, Porosity, Tensile Strength and Young Modulus for the different aerogels; Table S2. Density, Specific Tensile Strength (TS) and Young’s Modulus (YM) of the aerogels.
