# Supplementary material for: Cellulose Nanofiber-Based Aerogels from Wheat Straw: Influence of Surface Load and Lignin Content on Their Properties and Dye Removal Capacity
Source: Biomolecules. 2022 Jan 29;12(2):232. doi: 10.3390/biom12020232 (PMC8961610; doi:10.3390/biom12020232)
Supplement: Supplementary file 1 [file biomolecules-12-00232-s001.zip › biomolecules-1550986-supplementary.pdf]

SUPPLEMENTARY

Table S1. Results in Density, Porosity, Tensile Strength and Young Modulus for the different aerogels.

Treatment	Consistency	Density (mg/cm^3)	Porosity	Tensile Strength (kPa)	Young Modulus (kPa)
LCNF-Mec	**0,2**	7.46±1.94	99.61±0.03	23.44±3.56	230±20
	**0,4**	10.10±0.34	99.32±0.03	30.92±0.93	458±32
	**0,6**	14.27±0.28	99.06±0.07	46.70±4.41	418±1
	**0,8**	16.45±1.68	98,94±0.17	67.87±5.92	526±47
CNF-Mec	**0,2**	4.87±0.77	99.66±0.05	17.24±0.94	466±48
	**0,4**	7.60±1.02	99.83±0.24	18.80±0.45	413±160
	**0,6**	9.63±0.71	99.57±0.02	35.00±3.19	354±6
	**0,8**	13.43±1.19	99.24±0.08	61.58±1.93	646±138
LCNF-TO3	**0,2**	5.13±0.54	99.70±0.09	18.87±2.67	227±40
	**0,4**	6.23±0.32	99.79±0.28	19.36±0.24	259±43
	**0,6**	9.28±2.63	99.33±0.05	49.08±14.26	363±65
	**0,8**	10.76±1.39	99.20±0.00	42.27±13.60	389±91
CNF-TO3	**0,2**	3.96±0.15	99.76±0.05	16.64±3.37	298±190
	**0,4**	6.61±0.28	99.59±0.04	39.76±0.49	242±93
	**0,6**	9.67±0.62	99.34±0.03	34.86±1.89	266±38
	**0,8**	12.79±0.53	99.22±0.02	61.15±5.44	473±94
LCNF-TO5	**0,2**	4.96±0.63	99.75±0.17	20.06±2.31	277±28
	**0,4**	6.05±0.06	99.49±0.07	17.79±2.08	259±62
	**0,6**	9.77±0.86	99.36±0.05	33.75±12.59	270±77
	**0,8**	12.38±0.90	99.10±0.08	54.67±4.08	577±61
CNF-TO5	**0,2**	4.28±0.56	99.74±0.01	13.87±0.90	160±1
	**0,4**	5.71±0.93	99.55±0.02	18.23±0.60	167±20
	**0,6**	8.66±1.15	99.35±0.04	36.92±7.42	421±111
	**0,8**	13.85±3.35	99.15±0.04	61.00±2.63	484±101
LCNF-TO10	**0,2**	3.55±0.79	99.71±0.03	20.65±1.02	214±60
	**0,4**	6.03±0.58	99.62±0.06	26.32±2.80	274±29
	**0,6**	9.82±0.50	99.42±0.08	38.76±9.22	325±13
	**0,8**	11.59±0.24	99.08±0.22	51.80±1.41	536±13
CNF-TO10	**0,2**	3.82±0.55	99.75±0.04	16.43±0.32	136±78
	**0,4**	5.47±0.33	99.63±0.02	17.15±0.73	204±55
	**0,6**	8.63±0.68	99.42±0.05	29.25±2.75	286±70
	**0,8**	12.29±1.02	99.18±0.00	56.84±1.86	525±122

Table S2. Density, Specific Tensile Strength (TS) and Young´s Modulus (YM) of the aerogels.

Treatment	Consistency	Density (mg/cm³)	Specific TS (kPa·cm³/mg)	Specific YM (kPa·cm³/mg)
LCNF-Mec	**0,2**	7.46±1.94	3.41±0.23	33.45±2.87
	**0,4**	10.10±0.34	3.06±0.10	45.35±1.23
	**0,6**	14.27±0.28	3.27±0.31	29.29±0.04
	**0,8**	16.45±1.68	4.13±0.36	31.95±2.87
CNF-Mec	**0,2**	5.31±0.14	3.54±0.15	95.78±9.89
	**0,4**	7.60±1.02	2.47±0.06	54.36±21.01
	**0,6**	9.63±0.71	3.63±0.33	36.67±0.65
	**0,8**	13.43±1.19	4.58±0.14	48.07±10.29
LCNF-TO3	**0,2**	5.13±0.54	3.68±0.52	44.24±7.74
	**0,4**	6.23±0.32	3.10±0.04	41.50±6.94
	**0,6**	9.28±2.63	5,29±1.54	39.15±2.36
	**0,8**	10.76±1.39	3.93±1.26	36.10±8.48
CNF-TO3	**0,2**	3.96±0.15	4.20±0.85	75.31±47.89
	**0,4**	6.61±0.28	6.02±0.56	36.66±14.19
	**0,6**	9.67±0.62	3.60±0.20	27.52±3.98
	**0,8**	12.79±0.53	4.78±0.43	37.01±7.38
LCNF-TO5	**0,2**	4.96±0.63	4.05±0.05	55.80±4.56
	**0,4**	6.05±0.06	2.94±0.34	42.75±10.24
	**0,6**	9.77±0.86	3.45±1.29	27.65±7.92
	**0,8**	12.38±0.90	4.42±0.33	46.56±4.91
CNF-TO5	**0,2**	4.28±0.56	3.24±0.21	37.36±0.17
	**0,4**	5.71±0.93	3.19±0.11	29.18±3.52
	**0,6**	8.66±1.15	4,26±0.86	48.66±12.84
	**0,8**	13.85±3.35	4.40±0.19	34.94±7.26
LCNF-TO10	**0,2**	3.55±0.79	5.82±0.29	60.23±16.97
	**0,4**	6.03±0.58	4.70±0.05	45.48±4.81
	**0,6**	9.82±0.50	3.95±0.94	33.12±2.58
	**0,8**	11.59±0.24	4.47±0.12	46.30±1.08
CNF-TO10	**0,2**	3.82±0.55	4.47±0.23	49.60±19.72
	**0,4**	5.47±0.33	4,01±1.38	36.69±9.32
	**0,6**	8.63±0.68	5.02±1.99	44.86±8.46
	**0,8**	12.29±1.02	4.73±0.02	49.78±3.75
